# Impact of Genetic Testing Using Gene Panels, Exomes, and Genome Sequencing in Romanian Children with Epilepsy

**DOI:** 10.3390/ijms26104843

**Published:** 2025-05-19

**Authors:** Iulia Maria Sabau, Iuliu Stefan Bacos-Cosma, Ioana Streata, Bogdan Dragulescu, Maria Puiu, Adela Chirita-Emandi

**Affiliations:** 1 Doctoral School, Victor Babes University of Medicine and Pharmacy, 300041 Timisoara, Romania; 2 Regional Center of Medical Genetics Timis, Emergency Clinical Hospital for Children “Louis Turcanu”, 300011 Timisoara, Romania; adela.chirita@umft.ro; 3 Dr. Bacos Cosma Medical Center, Pediatric Neurology, 307200 Timisoara, Romania; 4 Laboratory of Human Genomics, University of Medicine and Pharmacy of Craiova, 200638 Craiova, Romania; 5 Regional Centre of Medical Genetics Dolj, Emergency Clinical County Hospital Craiova, 200642 Craiova, Romania; 6 Communications Department, Politehnica University Timisoara, 300006 Timisoara, Romania; 7 The Genomics Research and Development Institute, 020021 Bucharest, Romania; 8 Department of Microscopic Morphology, Genetics Discipline, Center of Genomic Medicine, “Victor Babes” University of Medicine and Pharmacy, 300041 Timisoara, Romania

**Keywords:** next generation sequencing, pediatric epilepsy, diagnostic yield, logistic regression, NGS testing

## Abstract

Epilepsy is a prevalent neurological condition, having a wide range of phenotypic traits, which complicate the diagnosis process. Next-generation sequencing (NGS) techniques have improved the diagnostics for unexplained epilepsies. Our goal was to evaluate the utility and impact of genetic testing in the clinical management of pediatric epilepsies. In addition, we aimed to identify clinical factors that could predict a genetic diagnosis. This was a retrospective study of 140 pediatric patients with epilepsy with or without other neurological conditions that underwent NGS testing (multigene panel, WES = whole exome sequencing and/or WGS = whole genome sequencing). A comparison between genetically diagnosed versus non-diagnosed children was performed based on different clinical features. Univariate and multivariate logistic regression analysis was performed to identify clinical predictors of a positive genetic diagnosis. Most children underwent gene panel testing, while 30 had exome sequencing and 3 had genome sequencing. The overall diagnostic yield of genetic testing was 28.6% (40/140) for more than 28 genes. The most frequently identified genes with causative variants were *SCN1A* (n = 4), *SCN2A* (n = 3), *STXBP1* (n = 3), *MECP2* (n = 2), *KCNQ2* (n = 2), *PRRT2* (n = 2), and *NEXMIF* (n = 2). Significant predictors from the logistic regression model were a younger age at seizure onset (*p* = 0.015), the presence of intellectual disability (*p* = 0.021), and facial dysmorphism (*p* = 0.049). A genetic diagnosis led to an impact on the choice or duration of medication in 85% (34/40) of the children, as well as the recommendation for screening of comorbidities or multidisciplinary referrals in 45% (18/40) of children. Epilepsy is a highly heterogeneous disorder, both genetically and phenotypically. Less than one third of patients had a genetic diagnosis identified using panels, exomes, and/or genomes. An early onset and syndromic features (including global developmental delay) were more likely to receive a diagnosis and benefit from optimized disease management.

## 1. Introduction

Epilepsy is a frequent neurological condition, with a global prevalence of 0.64% [1]. There is high genetic and phenotypic heterogeneity in epilepsy, which can make the diagnostic process more difficult [2,3]. According to current estimates, genetic factors contribute to 70–80% of epilepsy cases [4]. Genetic epilepsies can be broadly divided into rare monogenic epilepsies caused by single genetic variations and common epilepsies, characterized by a complex architecture of a multifactorial inheritance (multiple genetic variants together with environmental factors) [5,6].

Next-generation sequencing (NGS) improved the field of epilepsy genetics, with proven benefits in diagnosis, genetic counseling, and therapeutic approaches [7]. A genetic diagnosis in a patient with epilepsy can directly impact the choice of antiseizure medication, the eligibility for clinical trials, the cost of additional tests, the genetic testing for the relatives at risk, and the reproductive decision making [3].

The diagnostic yield of genetic tests for epilepsy can vary. A systematic review and meta-analysis that included 154 studies reported an overall diagnostic yield for genetic testing of 17% (48% for genome sequencing, 25% for exome sequencing, and 19% for gene panels) [8]. Stefanski A et al. in their systematic evidence review reported a 24% diagnostic rate for epilepsy, with the cohort of patients with intellectual disability and early-onset seizures having a higher diagnostic yield [9].

In Romania, NGS became available as part of the diagnostic workup relatively recently. In 2022, Riza et al. presented a case series of 36 Romanian patients with the early-onset developmental and epileptic encephalopathy (DEE) phenotype with an overall diagnostic rate of molecular genetic testing of 25% [10]. However, there is still a need for the evidence of utility and impact of genetic testing in the clinical management of epilepsy [8].

In our study, we assessed the utility of gene panels, exomes, and genome sequencing to diagnose the genetic etiology of Romanian children with epilepsy, with or without other neurological impairments. We aimed to evaluate the diagnostic yield of different genetic testing methods, as well as the impact on clinical management and the practice of precision medicine in the chosen cohort. In addition, we sought to assess the clinical predictors of a genetic diagnosis in NGS epilepsy testing.

## 2. Results

### 2.1. Patients’ Characteristics

The cohort consisted of 140 patients (male-to-female ratio 1:1) with a diagnosis of epilepsy/symptoms of seizures/modified EEG pattern with or without other neurological impairments, for which NGS genetic testing (multigene panels, WES, or WGS) was performed. The patients’ median age at the onset of seizures was 2.5 years (interquartile range IQR = 0.8–4.0). The median difference between the time at seizure onset and time of genetic testing was 2.1 years (IQR = 0–5.9). The age at seizure onset was statistically lower in the group of patients with a disease-causing variant compared to those without (*p* = 0.009), as well as the age at the time of genetic testing (*p* = 0.002). An epilepsy syndrome diagnosis, according to the 2022 ILAE classification [11], in all patients was not possible, as the seizure onset in categories of childhood onset, variable age onset, or idiopathic generalized epilepsy, could not be performed precisely; 35% of patients (n = 49) had diagnosed electroclinical epilepsy syndrome, out of which the most frequent included West syndrome (n = 7, 14.3%), genetic epilepsy with febrile seizures plus (n = 6, 12.2%), Dravet syndrome (n = 3, 6.1%), self-limited epilepsy with centrotemporal spikes (n = 3, 6.1%), and Lennox–Gastaut (n = 2, 4.1%). Epileptic encephalopathy or developmental and epileptic encephalopathy was present in 24 patients (17.0%). Fourteen patients (10%) had drug resistant epilepsy. Further details about patients’ phenotyping are available in Table 1.

### 2.2. Genetic Findings

**Figure 1:**
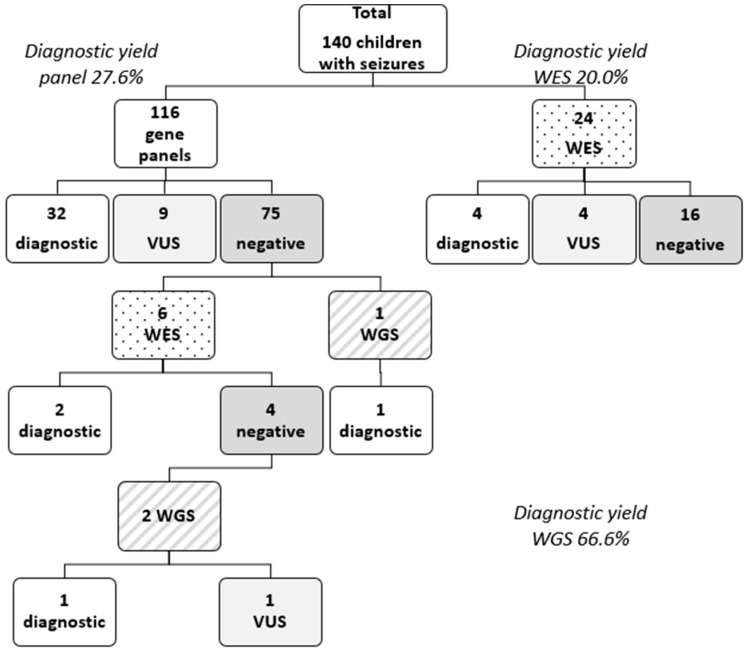
Patient genetic testing and diagnostic flowchart. Legend: VUS = variant of unknown significance; WES = whole-exome sequencing; WGS = whole-genome sequencing.

Out of the 140 patients, 116 patients underwent NGS gene panel testing, 30 patients underwent exome sequencing (19 solo WES, 10 trio WES, 1 quad WES), and 3 patients underwent WGS. A genetic etiology for epilepsy was identified in 40 patients, leading to an overall diagnostic yield of 28.6%. A positive result through multigene panel testing was obtained in 32 patients (27.6% diagnostic yield for multigene panel testing). From the subset of patients on which exome testing was performed, pathogenic or likely pathogenic variants were identified in 6 of them (20% diagnostic yield for exome sequencing). The WGS diagnostic yield was higher 66.6% (2/3) (see Figure 1 Patient diagnostic flowchart). However, most of the children from this cohort lacked an identifiable genetic diagnosis (n = 100, 71%). Fourteen unresolved cases (10%) are still under observation and need a further reevaluation of the VUS.

Information about familial segregation was obtained for 52 children only (37.1%), mostly due to a lack of family compliance. However, family screening enabled variant reclassification from a variant of uncertain significance to likely pathogenic or a confirmation that variants were in trans in 10 children, offering an additional diagnostic yield for genetic testing (+7.1%).

The most frequently identified genes with pathogenic or likely pathogenic variants were *SCN1A* (n = 4), *SCN2A* (n = 3), *STXBP1* (n = 3), *MECP2* (n = 2), *KCNQ2* (n = 2), *PRRT2* (n = 2), and *NEXMIF* (n = 2). A description of the causative genes according to the NCBI Gene database [12] is presented in the Supplementary File. The causative genes identified in this study encompassed 28 causative genes, of which 15 were primary epilepsy genes (*CACNA1A*, *KCNC1*, *KCNH5*, *KCNQ2*, *NRXN1*, *PRRT2*, *SCN1A*, *SCN2A*, *SCN8A*, *SLC2A1*, *STXBP1*, *SYNGAP1*, *TSC1*, *TSC2*, *WWOX*) and 13 were syndromic epilepsy genes or syndromes (Phelan–McDermid Syndrome/22q13.33 deletion syndrome—*ARSA*, *ALG12*; chromosome 16p12.2p11.2 deletion syndrome—*CLN3*, *EIF2B5*, *MECP2*, *MT-CYB*, *NEXMIF/KIAA2022*, *NFIA*, *NR2F1*, *P4HTM*, *PPP2R5D*, *PTCD3*, *PURA*, *RAI1*, *UBE3A*). In relation to the gene disease mechanism, from the total of 28 causative genes, 10 were epilepsy genes (*CACNA1A* coding a calcium channel, *SCN1A*, *SCN2A*, and *SCN8A* coding sodium channels, *KCNQ2* and *KCNC1* coding potassium channels, *SLC2A1* coding a glucose transporter, *STXBP1* coding a protein involved in membrane trafficking, *WWOX* coding an enzyme, and the *PRRT2* gene coding a transmembrane protein), 2 genes (*TSC1* and *TSC2* causing Tuberous Sclerosis) were neurodevelopment-associated genes, 7 genes were epilepsy-related genes (*UBE3A*, *PPP2R5D*, *PURA*, *MECP2*, *ALG12*, *ARSA*, *CLN3*), and 6 genes can be classified as potentially epilepsy-associated genes (*KCNH5*, *NEXMIF*, *NFIA*, *P4HTM*, *PTCD3*, *EIF2B5*).

Most of the causative variants were in a heterozygous state (35/40), four variants were in a compound heterozygous state and one mitochondrial variant had 5.3% heteroplasmy in the blood. A muscle biopsy was not accepted by the family for diagnosis. Among the positive results, the mode of inheritance of the causative genes was predominantly autosomal dominant (32/40), 4/40 had autosomal recessive inheritance, 3 variants were associated with dominant X-linked disorder (*MECP2* gene and *NEXMIF* gene), and the *MT-CYB* variant had a mitochondrial mode of inheritance. Most of the disease-causing variants were point mutations/single nucleotide variants (SNVs; n = 32/40, 80%). Classifying SNVs by molecular consequence, most of them were missense variants (20/32), five were nonsense, four were frameshift, and two were stopgain. The NGS multigene panel testing identified eight children with pathogenic/likely pathogenic CNVs or indels: five children with a deletion of the entire coding sequence, one variant consisting of a deletion of exons 7–18 in the *NRXN1* gene, one deletion of 12 bp of the *STXBP1* gene, and one indel/copy number gain of exon 5 of the *WWOX* gene. For three of the previously mentioned eight children, a SNParray or array CGH was performed to confirm the deletion.

Comparing the proportion of children with a disease-causing variant with those without based on different phenotypic subsets, a statistically significant difference was observed for the presence of the following: EE/DEE (*p* = 0.000), age at genetic testing under 2 years (*p* = 0.000), developmental delay (*p* = 0.000), stationary evolution (*p* = 0.000), speech delay (*p* = 0.001), stationary evolution (*p* = 0.002), facial dysmorphism (*p* = 0.008), age at seizure onset below 2 years (*p* = 0.009), intellectual disability (*p* = 0.014), autism spectrum disorder (*p* = 0.035), female gender (*p* = 0.040), as well as antiepileptic monotherapy (*p* = 0.043). A high genetic diagnostic yield was obtained for the subset of patients with EE/DEE 15/24 (62.5%), followed by facial dysmorphism (59%), an age at genetic testing under 2 years 10/17 (58.8%), stationary evolution 20/37 (54.1%), autism spectrum disorder 13/28 (46.4%), an age at onset of seizures under 2 years 27/60 (45.0%), intellectual disability 33/74 (44.6%), and developmental delay 32/74 (43.2%).

### 2.3. Impact of Genetic Diagnosis on Clinical Management

Identifying a genetic diagnosis led to an impact in the choice or duration of medication in 85% of the diagnosed patients (n = 34/40). Multidisciplinary management was influenced by the genetic diagnosis in 45% of positive cases (n = 18/40). A genetic diagnosis led to the recommendation of a ketogenic diet in 17.5% of the diagnosed cases (n = 7/40). One case (2.5%) was referred for a clinical trial. Table 2 summarizes the impact of a genetic diagnosis divided into the four categories mentioned above, showing the causative genes/syndromes in each category. All patients with a causative genetic variant received genetic counseling. More than half (52.5%) of the diagnosed patients had an impact in three categories (n = 21/40), while 37.5% (n = 15/40) had an impact in two categories and 7.5% (n = 3/40) had an impact in four categories.

### 2.4. Clinical Predictors of a Genetic Diagnosis

**Figure 2:**
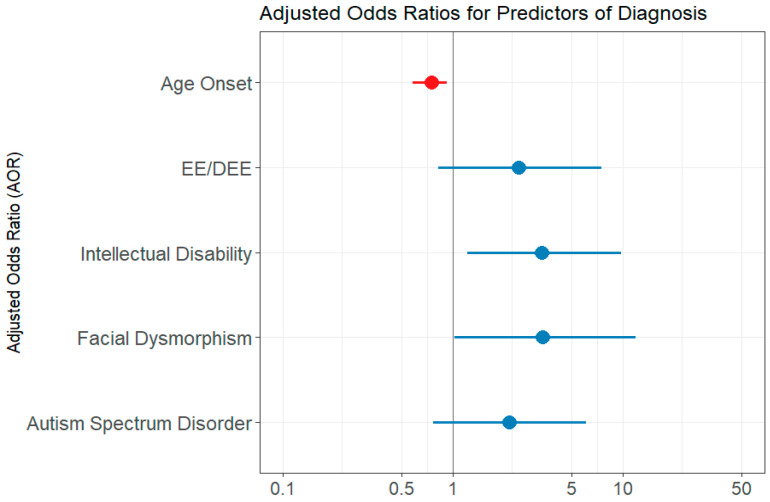
Multivariate logistic regression model evaluation—forest plot of adjusted odds ratio (AOR). EE/DEE = epileptic encephalopathy/developmental and epileptic encephalopathy; ID = intellectual disability; DD = developmental delay; ASD = autism spectrum disorder. The forest plot displays multivariate logistic regression results examining associations between clinical features and the likelihood of a molecular diagnosis. Red points represent predictors with AOR < 1, indicating an inverse relationship, while blue points represent predictors with AOR > 1, indicating a direct association. The horizontal lines indicate 95% confidence intervals. Predictors are considered statistically significant if their 95% confidence interval does not include 1.

The univariate logistic regression analysis showed the following predictor variables as statistically significant: age at seizure onset, presence of EE/DEE, presence of intellectual disability, developmental delay, autism spectrum disorder, facial dysmorphism (Table 3). The variable number of genes included in the test performed did not reach statistical significance (*p* = 0.856). However, when computing the multivariate regression model, which first included all of the significant variables from the univariate analysis, each of the variables became a weaker predictor and did not reach statistical significance. We assumed that the variables under investigation contained resembling information regarding the outcome and that not all of them are needed to capture the information [13]. Indeed, when we assessed the correlation between age at seizure onset and the presence of EE/DEE, we found a significant strong negative correlation, using both the Kendall test (*p*= 0.003) and Spearman test (*p* = 0.018). Developmental delay and intellectual disability variables were also found to be intercorrelated (Kendall test *p* = 0.0183). Intellectual disability was also correlated with the presence of EE/DEE (Kendal test *p* = 0.007, Spearman test *p* = 0.007). The final multivariate regression model included the following variables: age at epilepsy onset, EE/DEE, intellectual disability, facial dysmorphism, and autism spectrum disorder. The AIC value was 136.85. Features predictive of a genetic diagnosis were the age at seizure onset, intellectual disability, and facial dysmorphism. Autism spectrum disorder and EE/DEE did not reach statistical significance (Table 3 and Figure 2). The chi-squared verisimilitude test revealed a *p*-value of 0.003, showing that the model is statistically valid. In addition, the Hoslem–Lemeshow concordance test showed that the model is good to describe the data (*p* = 0.964). The training accuracy through cross-validation was 0.74, and the predictive accuracy was 0.88.

## 3. Discussion

This retrospective study evaluated 140 patients with epilepsy with or without other neurological conditions that underwent NGS testing. In 40 patients, pathogenic or likely pathogenic genetic variants were identified according to ACMG guidelines. The overall diagnostic yield was 28.6%, while gene panels had a diagnostic yield of 27.6% and exome sequencing had 20.0%. The highest diagnostic yield was found for genome sequencing (66.6%); however, only three children underwent WGS. Notably, six children for whom a gene panel was performed subsequently underwent WES. This is consistent with other previous studies, including a recent systematic review, which reported a 19% diagnostic yield for gene panel testing, 24% for exome sequencing, and 48% for genome sequencing in a cohort of 31,000 patients [8]. A. Stefanski et al., in their systematic review, reported an overall 24% diagnostic yield for sequencing testing (panels and exome) in epilepsy from 72 different studies [9]. Indeed the most comprehensive genetic test in epilepsy remains WGS, having the highest diagnostic yield of 48% in patients with epilepsy, as well as other neurodevelopmental disorders [8]. WGS has the advantage of detecting sequence variants, CNVs, and repeat expansion variations; however, it is not covered by the national insurance and interpretation is often hampered due to limited knowledge about the non-coding genome [14] On the other hand, multigene panel testing in epilepsies has the advantage of a lower cost and no incidental findings compared to wider testing; the drawbacks of panel testing in epilepsy might be that the newly discovered genes are not included in the panel. Furthermore, gene panels report a higher number of VUSs (depending on laboratory protocols), as panels are not usually performed in trio [14]. The cost-effectiveness of WES in the clinical practice of epilepsy has been proven before, leading to an overall reduction of costs due to shortening the time to diagnosis [15,16].

The diagnostic yield of genetic testing in epilepsy may vary, depending not only on the test performed, but also on the phenotype. For gene panels and WES, for example, the diagnostic yield may be highest for patients with early-onset DEE, ranging from 34% to 50% [14]. For multigene panels, the diagnostic yield is around 19%, but it might reach 54% when epilepsy is present along with structural brain abnormalities [8]. According to another study, patients with seizure onset before the age of 2 years had a 34% diagnostic yield using gene panels, whereas those with seizure onset after the age of 2 years had a 4% yield [17]. Other studies showed that the presence of developmental delay or intellectual disability may be predictive of a positive genetic diagnosis [18,19]. Rochtus et al. reported the highest diagnostic rate in patients with DEE (44%) [20]. In this cohort, we obtained a statistically significant diagnostic yield for the subset of patients that had developmental and/or epileptic encephalopathy, facial dysmorphism, developmental delay, seizure onset under 2 years, age at genetic testing under 2 years, intellectual disability, and autism spectrum disorder compared to those without. These findings suggest that patients with epilepsy and these characteristics should be swiftly referred to NGS testing to prevent diagnostic delays.

Although the panels had a variable number of genes included, this was not statistically correlated with the diagnostic yield in this study (logistic regression). The fact that the number of genes in the panel is not directly related to its yield has been previously highlighted [4,21,22]. Symonds et al. [23] concluded that *SCN1A*, *KCNQ2*, *CDKL5*, *SCN2A*, and *STXBP1* are the genes that have been implicated in the most NGS research to date. Therefore, the number of frequently mutated genes included in the panel (not the total number) is thought to be a good indicator of the diagnostic yield. In addition, the appropriate selection of patients is an important aspect to be considered as highlighted by Blazekovic et al. [22]. The diagnostic yield of WES was 40% in a cohort of 125 patients of whom 70% had development and epileptic encephalopathy [20].

This study showed high phenotypic heterogeneity in epilepsy. The median age at the onset of seizures in the cohort (2.5 years) was statistically lower when comparing the proportion of patients with causative variants with those without. This is in line with other studies showing that the age at seizure onset is lower in positive cases [21,24]. Based on the seizure type, 38.8% of patients in this cohort had focal seizures, 29.5% had generalized seizures, and 31.8% had mixed (focal and generalized) seizures. There was no statistical significance regarding the type of seizure and the outcome of a positive result of genetic testing. However, statistical significance was reached if the patient had EE/DEE, with the highest diagnostic yield being achieved in the subset of patients with these characteristics (*p* = 0.0001, diagnostic yield = 62.5%).

In this cohort, forty causative variants were identified in 28 different genes, showing high genetic heterogeneity. As a result of the advancement in genetic testing availability and collaborative studies, an increasing number of genes linked to epilepsy has been discovered [25]. Indeed, a recent paper has curated a list of 926 genes that are associated with monogenic disorder involving epilepsy [26]. As Dunn et al. stated, genetic epilepsies can be categorized into two categories: primary genetic epilepsies and epilepsies as part of the symptoms of a genetic neurological disorder [4,27]. Jie Wang et al., in their review [25], identified 977 genes associated with epilepsy and classified them into four categories: 84 primary epilepsy genes, 74 genes associated with epilepsy and brain-developmental malformations, 536 epilepsy-related genes, and 284 genes that require further validation. Classifications of the causative genes identified in this study were made based on the reviews mentioned above and are presented in the results section. Another aspect to consider in terms of genetic heterogeneity refers to the type of genetic variation; 80% (32/40) of the total disease-causing variants were point mutations/single nucleotide variants. However, 20% (8/40) were copy number variants or indels. This highlights the importance of NGS bioinformatic pipelines to call for CNV events. It has been previously shown that copy number alterations have a significant role in clinical epilepsy [28,29]. A large genome-wide and phenome-wide association study of more than 700,000 individuals evaluated the importance of CNVs in epilepsy and seizure-associated disorders and identified 25 significant genome-wide loci, out of which 22 were novel; in addition, phenome-wide association analysis between individual CNVs and HPO terms showed, for six CNVs, 19 significant associations [30].

Although establishing a genetic diagnostic in monogenic epilepsy is often quite difficult, understanding the pathophysiological mechanisms may enable the use of precision medicine, as well as treatment optimization [21]. Ion channel diseases are a good example to understand the difference between gain-of-function versus loss-of-function variants when making treatment decisions [31]. In Dravet Syndrome caused by *SCN1A*-loss-of-function variants, sodium channel blockers should be avoided [31]. However, gain-of-function variants in the *SCN2A* gene are known to respond well to sodium channel blockers [31]. There are also other examples including retigabine in loss-of-function variants in *KCNQ2* and *KCNQ3* genes [32], carbamazepine in *PRRT2*-related disorders [33], and a ketogenic diet in *SLC2A1* [33], among others. In addition, a genetic diagnosis for epilepsy may limit the need for additional tests, guide genetic counseling, inform the prognosis, or identify potential comorbidities [34]. Several studies evaluated the impact in the medical management of a genetic diagnosis in epilepsy [35,36,37,38] Haviland I et al. [7] reported an individualized direct medical impact in 72.4% of patients with a diagnostic result, with 45.4% of patients having an impact on treatment. In this cohort, 85% of the diagnosed patients had an impact in the choice or duration of the antiseizure medication. Two of the diagnosed individuals with a drug impact achieved seizure freedom (16p12.2p11.2 deletion syndrome, and the child with causative variant in *KCNH5*). However, six children with causative variants in the genes *SCN2A*, *PRRT2*, *NEXMIF*, *SCN1A*, and *SYNGAP1* had refractory epilepsy. A few examples of the drug impact of the genetic diagnosis in this cohort include the avoidance of valproic acid in the case of MELAS syndrome due to possible toxicity of mitochondria [39], the administration of Everolimus in *TSC1* Tuberous Sclerosis Complex [40], the recommendation of cannabidiol in developmental and epileptic encephalopathy *SYNGAP1* [41], and the administration perampanel in NR2F1-related epilepsy [42]. Furthermore, negative genomic findings could also be considered helpful. It was difficult to assess the impact of negative results in this retrospective cohort; however, we cannot rule it out. While a negative result does not rule out all genetic contributions, it can provide some reassurance that a major known pathogenic variant was not found. It may prompt clinicians to explore alternative diagnoses, such as structural, metabolic, or autoimmune causes [43].

Prediction models are often used to help healthcare providers in the application of diagnostic tests [13]. A logistic regression model estimates the association between one or more independent variables (also called, predictors) with a binary dependent variable (also called, the outcome variable) [44]. In this study, univariate and multivariate logistic regression analysis was used to identify predictors for a positive genetic test result. Haviland et al. made a similar analysis, showing that an age at epilepsy onset under 2 years, focal motor seizures, developmental delay, and malformation of brain development are statistically significant predictors for the chosen outcome [7]. Benevides et al. [45] performed a multivariate analysis to determine predictors of a positive genetic diagnosis, showing that the first seizure in the context of fever and hypotonia were positively corelated with a genetic etiology, while atonic seizures were negatively associated. Wong et al. [46] used a classification and regression tree analysis to identify the phenotypic features associated with a genetic diagnosis in a cohort of 316 patients with neurodevelopmental disorders, showing that the female gender, history of motor delay, hypotonia, congenital heart disease, and early intervention were more likely to be associated with a genetic etiology. These studies show that prediction models based on phenotypic data could help clinicians to stratify patients for genetic testing.

While genetic testing in epilepsy led to a good diagnostic yield, there is still a high percentage of negative results in this cohort (71%). These unresolved genetic epilepsies may be due to several reasons, such as the following: complex polygenic inheritance, diseases that may be monogenic, but there are many genes that are not yet associated with a human disorder, the molecular defect could not be detected by the technique used (such as somatic mosaicism or complex structural rearrangements), the gene was not included in the panel, uninformative variant classification, such as VUS, due to limited data, the defect may be in the non-coding genome (regulatory elements, topologically associated domains, etc.). Potentially, the etiology of epilepsy might be uncovered by studying other omics (transcriptomics, epigenomics, or metabolomics) [43].

## 4. Material and Methods

### 4.1. Cohort Information

This retrospective study included pediatric patients diagnosed with epilepsy and/or other neurological disorders who underwent genetic testing between August 2019 and November 2023. The patients were referred to a tertiary care pediatric neurology clinic (Dr BACOS Cosma Iuliu Medical Center), with a minority being referred to the Regional Center of Medical Genetics Timis and Regional Center of Medical Genetics Dolj. All patients were examined by both a pediatric neurologist and a clinical geneticist.

The inclusion criteria were a clinical diagnosis of epilepsy, seizure-like symptoms or EEG (electroencephalogram) suggestive of epilepsy, and the availability of a genetic testing report (gene panels/WES/WGS). The exclusion criteria included patients who had epilepsy but did not undergo genetic testing, as well as those who underwent genetic testing but did not have a diagnosis of epilepsy, seizure symptoms, or EEG findings suggestive of epilepsy. A genetic testing decision was made on a case-by-case basis and initiated based on the neurologist’s or geneticist’s clinical judgment. In most cases, genetic testing was recommended after inconclusive imaging and EEG results. However, in some specific syndromic cases (e.g., suspected Tuberous Sclerosis Complex), genetic testing was used to confirm a suspected diagnosis based on clinical and imaging findings. Panel testing was most used as the first-tier test, with WES/WGS reserved for more complex or undiagnosed cases. However, patients who underwent panel testing were typically seen earlier in the study and often presented with more severe phenotypes. WES and WGS became more accessible later and were used for a broader spectrum of epilepsy types.

Data were retrospectively collected from the medical records of all patients, including demographic information, gender, age at genetic testing, age at epilepsy onset, associated comorbidities (intellectual disability, development delay, autism spectrum disorder, other comorbidities), seizure type, EEG findings, results of brain imaging (MRI-Magnetic Resonance Imaging or CT-Computed Tomography), and number of antiepileptic drugs used at last visit. The seizure type was subdivided into focal, generalized, focal, and generalized, with a special category of DEE [11]. The term DEE denotes “an epilepsy associated with developmental impairment that may be due to both the underlying etiology (development encephalopathy) and superimposed epileptic activity (epileptic encephalopathy)” [47]. In this cohort, individuals were considered as having DEE if they had a suspicion or confirmation of a genetic etiology, epilepsy onset <18 years, and an EEG pattern suggesting encephalopathy, as well as the presence of intellectual disability or development delay. We assessed whether the patient had drug-resistant epilepsy and a history of status epilepticus and whether the epilepsy was part of a genetic syndrome. To assess the severity of disease, the number of antiepileptic drugs and the frequency of seizures was reported (according to last medical visit). The frequency of seizures was classified into six categories: (a) one or fewer than one seizure per year, (b) one seizure per year, (c) 2–3 seizures per year, (d) monthly seizures, (e) one seizure per week, and (f) daily seizures. Drug-resistant epilepsy was defined as the “failure of adequate trials of two tolerated and appropriately chosen antiepileptic drugs schedules (whether as monotherapies or in combination) to achieve seizure freedom” [48]. Status epilepticus was defined considering revised guidelines from the Neurocritical Care Society as being a seizure lasting for five minutes or longer, presenting clinical and/or electrographic continuous seizure activity, or repeated seizure activity without recovery between episodes [49]. If possible, the epilepsy diagnosis was further specified according to the 2022 ILAE classification of epileptic syndromes [11]. Patients with idiopathic/benign epilepsies were included. The evolution of each patient’s disease was classified into three categories: favorable, stationary, and unfavorable/worsened evolution.

The study was approved by the Ethics Board of the Faculty of Medicine of University of Medicine and Pharmacy Victor Babes Timisoara (No. 64/8 November 2024). All patients included in the study gave their informed consent for anonymously using their clinical data and results of genetic testing for research purposes. This study was performed in accordance with the 1964 Declaration of Helsinki.

### 4.2. Genetic Testing

Prior to enrollment in the analysis, all the included patients underwent NGS genetic testing, either via Invitae Multigene Panel(s) testing (San Francisco, CA, USA), Blueprint Genetics WES (Espoo, Finland), or Centogene Whole Genome Sequencing (Rostock, Germany). All genetic tests included the detection of copy number variants/exonic deletions/exonic duplications. The number of genes included in the Invitae Epilepsy Panel varied from 133 to 964, depending on the time at genetic testing, as new genes were added to the panel throughout the study period. In some cases, depending on the phenotype and the genes available in the current epilepsy panel, one or more panels/genes were added. Besides the Invitae Epilepsy Panel, the additional panels/genes ordered by the clinician were as follows: Preliminary-evidence Genes for Epilepsy, Glycine Encephalopathy, FLNA gene, PTEN gene, RANBP2 gene, Tuberous Sclerosis Complex Panel, Rett and Angelman Syndromes and Related Disorders Panel, Supplemental Metabolic Newborn Screening Panel, Cerebral Palsy Spectrum Disorders Panel, Neurodevelopmental Disorders (NDD) Panel, Comprehensive Neurometabolic Disorders Panel.

Genetic variants were clinically interpreted considering the recommendation guidelines of the ACMG-AMP (American College of Medical Genetics and Genomics—Association for Molecular Pathology) published in 2015 [50]. Therefore, the genetic test result was categorized into one of the five ACMG-AMP categories: “pathogenic”, “likely pathogenic”, “uncertain significance”, “likely benign”, “benign”. Results were considered as disease-causing (positive) when identifying the presence of a pathogenic or likely pathogenic genetic variant: (1) in a heterozygous state associated with dominant conditions; (2) in a homozygous state or compound heterozygous state (in trans) associated with autosomal recessive conditions; and (3) in a hemizygous state associated with an X-linked recessive condition in males. All the other outcomes, including carrier results and variants of uncertain significance (VUS), were considered negative. In some cases of VUS that were highly suspected of pathogenicity based on the phenotype, we assessed the variant segregation in the patient’s family for a possible reclassification. Causative epilepsy genes identified were further classified based on existing classifications in the literature [4,25].

The clinical impact of genetic testing was evaluated in five categories: drug impact, dietary changes, clinical trials, multidisciplinary management, and genetic counseling. A direct impact on the choice of antiseizure medication was considered if the genetic diagnosis had an influence on the choice of the antiepileptic drug, had a specific drug contraindication, there was an influence regarding the duration of the medication, or there was a change in the medication. An impact in multidisciplinary management was considered if the genetic diagnosis led to recommendations for the screening of associated comorbidities or a referral for a multidisciplinary dispensary other than neurology.

### 4.3. Data Analysis

Clinical patient data were inserted into a Microsoft Excel sheet [51]. Further statistics and data analysis were performed using R-4.4.2 and R-Studio 2024.12.0+467 [52,53]. The diagnostic rate was computed as the number of genetically diagnosed patients in a specific subset (e.g., subset of patients with seizure onset under two years) and then reported as the percentage of the total number of patients in that category. Pearson’s chi-squared test statistic or a Fisher test were used to compare the proportion of the positive results versus negative results (R package stats, functions prop.test/fisher.test [52]). The Anderson normality test was used to asses whether the data had a normal distribution (function ad.test, R package nortest [54]. The Wilcoxon rank sum test was used to compare two independent continuous variables that did not follow a normal distribution (function wilcox.test). A logistic regression model was used to assess clinical factors predictive of a positive genetic diagnosis. Before creating the multivariate logistic regression model, univariate logistic regression was used to examine each predictor separately. A background selection approach was used to build the multivariate logistic regression model. The predictors in the final model were chosen based on the model’s Akaike information criterion (AIC) value and taking into consideration if there was a correlation between variables. The correlation of two variables that did not follow a normal distribution was assessed using the Kendall rank and Spearman rank correlation test (function cor.test). The evaluation of the logistic regression model included a quality evaluation of each predictor using the *p*-value and odds ratio, goodness of fit, and accuracy. A chi-squared verisimilitude test was used to check if the model was statistically validated (function lrtest, R package lmtest [55]. The Hosmer–Lemeshow test was used to check if the model was good to describe the data (function hoslem.test, R package ResourceSelection [56]. A forest plot was built to visualize the predictors ‘adjusted odds ratio of the model (function plot_model R package sjPlot [57]. The R package boot [58] was used to compute the model’s training and predicted accuracy. Statistical significance was defined as a value of *p* < 0.05.

## 5. Limitations

There are limitations to this observational retrospective study. First, the sample size was relatively small, which may have limited the statistical power. Perhaps, with higher a power rate, other predictors would have reached significance when evaluating the clinical predictors of a genetic diagnosis through genetic testing. Second, patient selection bias is possible given the evolving genetic testing practices over the study period. This study was conducted when the practice of genetic testing for epilepsy was evolving. In early years, genetic testing was most probably affected by selection bias; the most severely affected patients were proposed for genetic testing, which mainly consisted of multigene panel testing. On the other hand, in more recent years, a broader group of epilepsy patients underwent genetic testing, which consisted of exome sequencing. However, the Invitae laboratory regularly reevaluates variant classification and informs the physician and patient about reclassification. Even more, Invitae offers WES reanalysis every year and phenotypic data are requested to the physician. Test selection bias (for gene panel or WES) might be the reason for a lower diagnostic yield of exome sequencing compared to multigene panels. Another limitation would be that the study was performed on a Romanian population only, which may restrict its generalization. Nonetheless, this population has very few published studies investigating the genotype and phenotype in children with seizures. Regarding data collection, although all efforts were made to reduce missing data, some variable values remained incomplete. Epilepsy syndromes were classified using the ILAE 2022 [11] framework when possible. However, due to retrospective data collection and evolving diagnostic documentation, we could not systematically subcategorize all patients prior to testing. In addition, due to sample size constraints, we did not analyze benign or idiopathic epilepsies or developmental and epileptic encephalopathies separately, but it will be worth exploring in future studies. Furthermore, due to a lack of family compliance and other factors regarding regular visits and recommendations, segregation analysis was not performed on all children where necessary. A significant limitation of the study is that the impact of genetic testing was only evaluated in the patients with a genetic diagnosis. In addition, the numbers and percentages reported might be underestimated due to a lack of follow-up documentation, as some of the patients were followed elsewhere.

## 6. Conclusions

The genetic diagnosis of epilepsy in Romanian children was highly heterogeneous, both phenotypically and genetically, with 40 variants identified in 28 genes. Fewer than one third (28.6%) of patients had a genetic diagnosis identified using panels, exomes, and/or genomes, leading to changes in medical management, including treatment recommendations and surveillance for comorbidities. Early-onset and syndromic features (including global developmental delay) were more likely to receive a diagnosis and benefit from optimized disease management.

## Figures and Tables

**Table 1:** Clinical characteristics of 140 pediatric patients with epilepsy and comparison between patients with and without a disease-causing genetic variant.

Variables	Variable Categories	Total n = 140 (100%)	Diagnostic n = 40	Non-Diagnostic n = 100	*p*-Value
Test performed	Multigene Panel	116 (82.9%)	32 (80.0%)	85 (85.0%)	0.639
	WES	30 (21.4%)	6 (15.0%)	24 (24.0%)	0.345
	WGS	3 (2.1%)	2 (5.0%)	1 (1.0%)	1.000
Gender	Female	70 (50.0%)	26 (65.0%)	44 (44.0%)	**0.040**
Age at seizure onset	Median = 2.5 IQR = 0.8–4.0		Median = 1.0 IQR = 0.3–2.5	Median = 3.0 IQR = 1.3–6.0	**0.009**
	<2 years	60 (42.9%)	27 (67.5%)	33 (33.0%)	**0.000**
Age at genetic testing	Median = 5.0 IQR = 3.0–9.0		Median = 3.5 IQR = 1.8–6.0	Median = 6.0 IQR = 3.0–10.5	**0.002**
	<2 years	21 (15.0%)	12 (30.0%)	9 (9.0%)	**0.004**
Time from seizure onset to genetic testing	Median = 2.1 IQR = 0.0–5.9		Median= −1.2 IQR = (−3.9)–(−0.2)	Median= −1.7 IQR = (−4.8)–(−0.1)	0.837
Number of genes included in the panel	Median = 306.0 IQR = 192.0–320.0		Median = 306.0 IQR = 188.5–320	Median = 306.0 IQR = 274.5–320.0	0.405
Seizure type *	Focal	50/129 (38.8%)	12/35 (34.3%)	38/94 (40.4%)	0.665
	Generalized	38/129 (29.5%)	9/35 (25.7%)	29/94 (30.9%)	0.725
	Mixed	41/129 (31.8%)	14/35 (40.0%)	27/94 (28.7)	0.312
Status epilepticus	Present	10 (7.1%)	2 (5.0%)	8 (8.0%)	1.000
Frequency of seizures **	less than one seizure per year	46/112 (41.1%)	9/30 (30.0%)	37/82 (45.1%)	0.221
	1 seizure per year	23/112 (20.5%)	3/30 (10.0%)	20/82 (24.4%)	1.000
	2–3 seizures per year	12/112 (10.7%)	3/30 (10.0%)	9/82 (11.0%)	1.000
	monthly seizures	7/112 (6.3%)	3/30 (10.0%)	4/82 (4.9%)	1.000
	weekly seizures	9/112 (8.0%)	5/30 (16.7%)	4/82 (4.9%)	1.000
	daily seizures	15/112 (13.4%)	7/30 (23.3%)	8/82 (9.8%)	0.120
EE/DEE	Present	24 (17.1%)	15 (37.5%)	9 (9.0%)	**0.000**
Intellectual disability	Present	74 (52.9%)	33 (82.5%)	41 (41.0%)	**0.014**
Developmental delay	Present	74 (52.9%)	32 (80.0%)	42 (42.0%)	**0.000**
Speech delay	Present	80 (57.1%)	32 (80.0%)	48 (48.0%)	**0.001**
Autism spectrum disorder	Present	28 (20.0%)	13 (32.5%)	15 (15.0%)	**0.035**
Developmental regression	Present	11 (7.9%)	4 (10.0%)	7 (7.0%)	1.000
Drug-resistant epilepsy	Present	14 (10.0%)	6 (15.0%)	8 (8.0%)	0.340
Facial dysmorphisms	Present	17 (12.1%)	10 (25.0%)	7 (7.0%)	**0.008**
Congenital malformations	Yes	9 (6.4%)	4 (10.0%)	5 (5.0%)	1.000
Ataxia	Yes	7 (5.0%)	4 (10.0%)	3 (3.0%)	1.000
Ophthalmologic features	Yes	15 (10.7%)	7 (17.5%)	8 (8.0%)	0.180
Muscle tone	Abnormal	20 (14.3%)	8 (20.0%)	12 (12.0%)	0.340
Movement disorder	Present	12 (8.6%)	4 (10.0%)	8 (8.0%)	1.000
Motor deficit	Present	10 (7.1%)	1 (2.5%)	9 (9.0%)	1.000
Cerebral palsy	Present	2 (1.4%)	0 (0%)	2 (2.0%)	1.000
Behavioral disorder	Present	11 (7.9%)	5 (12.5%)	6 (6.0%)	0.345
Number of antiepileptic drugs at last visit ***	0	12/119 (10.1%)	1/31 (3.2%)	11/88 (12.5%)	1.000
	1	55/119 (46.2%)	9/31 (29.0%)	46/88(52.3%)	**0.043**
	2	34/119 (28.6%)	13/31 (41.9%)	21/88 (23.9%)	0.092
	3	8/119 (6.7%)	4/31 (12.9%)	4/88 (4.5%)	0.214
	4	10/119 (8.4%)	4/31 (12.9%)	6/88 (6.8%)	1.000
EEG findings ****	Pathological	107/117 (91.5%)	30/30 (100.0%)	77/87(88.5%)	0.118
Evolution *****	Favorable	53/94(56.4%)	10/32 (31.3%)	43/62 (69.4%)	**0.000**
	Stationary	37/94 (39.4%)	20/32 (62.5%)	17/62 (27.4%)	**0.002**
	Unfavorable	4/94 (4.3%)	2/32 (6.3%)	2/62 (3.2%)	1.000

EE/DEE = epileptic encephalopathy/developmental and epileptic encephalopathy; EEG = electroencephalogram; IQR = interquartile range; DY = diagnostic yield. * percent missing 7.9% (11/140), ** percent missing 20% (28/140), *** percent missing 15% (21/140), **** percent missing 16.4% (23/140), ***** percent missing 32.6% (46/140). Note: values are expressed as the total number of patients for the phenotypic trait taken into consideration. Percentages were rounded to one decimal for clarity. *p*-values were computed by comparing patients with a genetic diagnosis with those without. Statistical test used: Pearson’s Chi-squared test/Fisher’s exact test/Wilcoxon rank sum test. Significant *p*-values are in bold.

**Table 2:** Impact of genetic testing in patients with a genetic diagnosis.

Type of Genetic Testing Impact	Values from the 40 Children with a Genetic Diagnosis (%)	Causative Gene/Syndrome
Drug impact	n = 34 (85.0%)	*CACNA1A*, *EIF2B5*, *KCNC1*, *KCNH5*, *KCNQ2*, *MECP2*, *MT-CYB*, *NEXMIF(KIAA2022)*, *NR2F1*, *NRXN1*, *PPP2R5D*, *PRRT2*, *PURA*, *SCN1*, *SCN2A*, *SCN8A*, *SLC2A1*, *STXBP1*, *SYNGAP1*, *TSC1*, *TSC2*, *UBE3A*, *WWOX*
Ketogenic diet	n = 7 (17.5%)	*NR2F1*, *PPP2R5D*, *SCN2A*, *SLC2A1*, *STXBP1*, *SYNGAP*
Clinical trials	n = 1 (2.5%)	*EIF2B5*
Multidisciplinary dispensary	n = 18 (45.0%)	Phelan–McDermid syndrome, 16p12.2p11.2 deletion syndrome, *EIF2B5*, *MECP2*, *MT-CYB*, *NEXMIF(KIAA2022)*, *NFIA*, *NR2F1*, *NRXN1*, *P4HTM*, *PPP2R5D*, *PURA*, *RAI1*, *TSC1*, *TSC2*, *UBE3A*

**Table 3:** Univariate and multivariate logistic regression models of phenotypic features as predictors of a positive genetic diagnosis.

Variable	OR/Adjusted OR	95% Confidence Interval	*p*-Value
Univariate Logistic Regression Models			
Age Onset seizures	0.673	0.528–0.818	0.004
EE/DEE	6.066	2.419–16.047	0.036
ID	6.783	2.875–18.065	0.024
DD	5.523	2.408–13.988	0.018
ASD	2.728	1.147–6.478	0.022
Status epilepticus	0.605	0.088–2.553	0.537
Facial dysmorphism	4.428	1.567–13.187	0.005
Number of genes in the panel	1.001	0.998–1.002	0.856
Multivariate Logistic Regression Model			
Age onset	0.752	0.580–0.920	0.015
EE/DEE	2.438	0.824–7.502	0.111
Intellectual disability	3.331	1.223–9.759	0.021
Facial Dysmorphism	3.377	1.026–11.959	0.049
ASD	2.156	0.772–6.078	0.141

EE/DEE = epileptic encephalopathy/developmental and epileptic encephalopathy; ID = intellectual disability; DD = developmental delay; ASD = autism spectrum disorder.

## Data Availability

The data supporting the findings of this study are available in the Supplementary Materials. Additional information may be provided by the corresponding author upon reasonable request.

## Supplementary Materials

The following supporting information can be downloaded at: https://www.mdpi.com/article/10.3390/ijms26104843/s1.
